# Patient safety and staff psychological safety: A mixed methods study on aspects of teamwork in the operating room

**DOI:** 10.3389/fpubh.2022.1060473

**Published:** 2022-12-23

**Authors:** Dana Arad, Adi Finkelstein, Ronen Rozenblum, Racheli Magnezi

**Affiliations:** ^1^Health System Management Department, Bar-Ilan University, Ramat Gan, Israel; ^2^Patient Safety Division, Ministry of Health, Jerusalem, Israel; ^3^Department of Nursing, Jerusalem College of Technology, Jerusalem, Israel; ^4^Brigham and Women's Hospital, Boston, MA, United States; ^5^Harvard Medical School, Boston, MA, United States

**Keywords:** patient safety, psychological safety, operating room, teamwork, safety standards

## Abstract

**Objectives:**

To predict the amount of teamwork that takes place throughout a surgery, based on performing a preoperative safety standards (surgical safety checklist and surgical count) and to explore factors affecting patient safety and staff psychological safety during a surgery, based on interprofessional teamwork.

**Methods:**

This mixed methods study included quantitative and qualitative analyses. Quantitative data included 2,184 direct observations of surgical cases with regard to the performance of safety standards during surgeries in 29 hospitals, analyzed using multivariate binary logistic regressions. Qualitative data were obtained from an analysis of 25 semi-structured interviews with operating room (OR) clinicians and risk managers, using an inductive thematic analysis approach.

**Results:**

Analysis of the OR observations revealed that a lack of teamwork in the preoperative “sign-in” phase doubled the chances of there being a lack of teamwork during surgery [odds ratio = 1.972, 95% confidence interval (CI) 1.741, 2.233, *p* < 0.001] and during the “time-out” phase (odds ratio = 2.142, 95% CI 1.879, 2.441, *p* < 0.001). Consistent presence of staff during surgery significantly increased teamwork, by 21% for physicians and 24% for nurses (*p* < 0.05), but staff turnover significantly decreased teamwork, by 73% for physicians (*p* < 0.05). Interview data indicated that patient safety and staff psychological safety are related to a perception of a collaborative team role among OR staff, with mutual commitment and effective interprofessional communication.

**Conclusions:**

Healthcare organizations should consider the key finding of this study when trying to identify factors that affect teamwork during a surgery. Effective preoperative teamwork positively affects intraoperative teamwork, as does the presence of more clinicians participating in a surgery, with no turnover. Other factors include working in a fixed, designated team, led by a surgeon, which functions with effective interprofessional communication that promotes patient safety and staff psychological safety.

## Introduction

Patient safety is an ongoing concern in operating rooms (OR) due to the complex work environment, a high level of stress, and vulnerable patients (1, 2); these factors can lead to the occurrence of errors and patient harm. Additionally, standard safety checks to prevent errors are sometimes omitted or not fully performed (3). Teamwork is a major component in the promotion of safety; however, most surgical teams include clinicians from various disciplines, with differing priorities, roles, backgrounds, and expertise (4). Although they share the goal of providing safe and successful surgical care (5, 6), they are susceptible to errors such as performing wrong-site surgery (2). Major errors in the OR, or surgical “Never Events” (such as wrong-site surgery and retained foreign items during surgery) are preventable, unjustifiable adverse events that should be reduced through quality improvement that involves better teamwork (7).

Effective teamwork is an essential component of safe surgery (8). Teamwork is defined as a dynamic process involving two or more healthcare professionals with complementary backgrounds and skills, sharing common health goals (9–13). A surgical team is defined as comprising “professionals of different disciplines, educational backgrounds, and experiences (who) must work interdependently in a dynamic, high-stakes environment” (14). Surgical outcomes are strongly dependent on communication and cooperation among the surgical team (15–18). Thus, ineffective teamwork is linked to poorer surgical outcomes for patients and reduced patient safety that can result in adverse events (19).

Teamwork is not only related to patient safety. Some of the factors that inhibit teamwork can be explained by the concept of staff psychological safety. Psychological safety represents a shared belief among a team that it is safe to engage in interpersonal risk-taking, this feeling being necessary for team learning and working toward a common goal (20). Generally, poorly defined tasks and a lack of resources lead to a poor sense of psychological safety, whereas leadership, trust among team members, and an ability to solve problems (21) engender an environment that fosters empowerment (22). Consistent with this notion, studies have shown that empowered and enhanced practice entails teamwork, communication, and supportive supervision, which is associated with improved team performance and to a lesser extent with patient outcomes (23).

Bates and Singh (24) described the importance of policies to prevent both previously known and unanticipated risks. Surgical safety standards promote and enable a sense of staff psychological safety during a surgery in order to prevent Never Events (25). The World Health Organization's surgical safety checklist and the use of surgical counts require collaboration between nurses and physicians, thereby encouraging intra- and inter-disciplinary teamwork (26).

In this study, we analyzed the effect of preoperative teamwork on intraoperative teamwork between physicians and nurses in relation to adherence to safety standards and staff turnover; we also evaluated the concepts of individual and team role definitions in relation to safety. We used a mixed methods design, because quantitative data can provide only a partial understanding of effective teamwork, while an analysis of qualitative data enabled us to refine and explain the quantitative results by exploring participants' views regarding teamwork and aspects of staff psychological safety (27).

## Methods

The current study used a triangulation, mixed methods convergence design to analyze teamwork in the OR (28). It included a retrospective cohort study that used data captured from observations of safety standards in the OR to predict the level of teamwork throughout a surgery; we also conducted purposive recruitment of individuals to participate in semi-structured interviews regarding their perceptions of safety in the OR (29).

### Participants

#### Quantitative dataset

Observers recruited by the Israeli Ministry of Health (MOH) observed the adherence to surgical safety standards during surgical cases in terms of quality control and patient safety assessments, in 29 general hospitals (based on the MOH criteria for a general hospital) in Israel between December 2018 and May 2021. Five large hospitals had >800 beds, 10 medium hospitals had 400–800 beds, and 14 small hospitals had <400 beds. Seven of the hospitals were in rural areas and 22 in urban areas.

#### Qualitative dataset

We interviewed 25 individuals, comprising OR clinicians (anesthesiologists, surgeons, and nurses with management positions who currently practice in ORs) and risk managers from general hospitals and the MOH, based on what we anticipated to be sufficient to achieve data saturation. Five risk managers were from the MOH, and 20 interviewees were clinicians and risk managers from eight hospitals (four large hospitals with >800 beds, two medium hospitals with 400–800 beds, and three small hospitals with <400 beds; five were in urban areas and three in rural areas).

### Data collection

#### Quantitative observations

We used data from 2,184 different surgical cases; the data were collected by the MOH using accepted guidelines for making direct observations. The direct observations were performed by trained observers on the performance of a surgical safety checklist and surgical counts throughout a surgery, based on international and national guidelines for their performance. The surgical cases observed were selected at random by the observers from the planned operations plan in each OR on the day of observation, taking care to not always observe the same team members. The observations were performed by physicians, medical students, nurses, or nursing students. All observers underwent simulation training for 8 h. To ensure observers were competent, observers with >5% discordance between their observation entries and the expected entries in the simulation were not allowed to perform the observations. For the purposes of our study, we chose items in the surgical safety checklist and surgical counts that represent teamwork throughout a surgery, as they require the mutual performance of more than one team member, for example, two nurses or a physician and a nurse, or the mutual performance of all team members present for a surgery (Appendix 1). In the surgical cases observed there were no observations involving the occurrence of Never Events.

#### Qualitative semi-structured interviews

The 25 interviews were conducted between September and December 2019 by one of the authors (DA). Participants were approached based on their professional position and the size and location of their hospital (Appendix 2). The interviews were audio recorded and the recordings were transcribed verbatim. Participants provided verbal consent to participate and received no compensation. The interviews were conducted in person at the participants' offices and lasted an average of 20 min.

Field notes were taken by one of the authors (DA) during and immediately after each interview, in which the interviewees described factors contributing to surgical errors and Never Events, and recorded any nonverbal reactions, such as anger or discomfort, during the interview.

### Analysis

#### Quantitative analysis

The statistical software package SPSS-25 was used to analyze the data captured during the observations. A multivariate logistic regression model was used to predict the level of teamwork during a surgery based on two measures: the level of preoperative teamwork as a predictor of teamwork during surgery and the effect of staff presence and turnover on teamwork.

#### Preoperative teamwork

The variable representing a lack of preoperative teamwork included seven items (Appendix 1), expressing the level of team collaboration when performing a surgical safety checklist during sign-in and time-out phases right before the beginning of a surgery. A lack of teamwork was defined as the number of items in which the team did not work together. We ranked the variable from 0 to 7 (where 0 represents the most teamwork and 7 represents the least).

#### Intraoperative teamwork

The variable representing intraoperative teamwork was created from four items performed during the second surgical count (Appendix 1). At that point, two nurses perform the surgical count together and include the surgeon in the process. A lack of teamwork was defined as the number of items on which the team did not work together. The variable was ranked from 0 to 4 (where 0 represents the most teamwork and 4 represents the least).

#### Staff presence and turnover

To evaluate the effect of staff turnover throughout a surgery on teamwork, we created two variables. The first evaluated the mean number of physicians (anesthesiologists and surgeons) and nurses participating in sign-in, time-out, and second surgical count throughout the surgery. The second evaluated the standard deviation (SD) of the number of physicians and nurses present during a surgery to represent staff entering and leaving the OR. For this measure, the higher the number, the higher the turnover (0 represents no change).

#### Qualitative analysis

The interviews analyzed factors that contribute to surgical Never Events in the OR. The interview guide (Appendix 2) was developed based on opinions from clinicians and risk management experts and the categories of contributing factors evolved inductively from the interviews. To test the interview guide, two pilot interviews with two participants were conducted, after which one question was omitted due to lack of relevance to the study. The data from the pilot study were added to the final analysis.

For the qualitative analysis, we used the six-phase inductive thematic analysis approach described by Braun and Clarke (30):

(1) Data familiarization—two investigators (DA and AF) independently read and re-read the transcripts to establish familiarity with the data and to search for possible meanings and patterns.
(2) Generating initial codes—the initial codes were independently generated from the data by two investigators (DA and AF) to generate topics of interest, following an inductive coding approach.
(3) Searching for themes—the various codes were sorted into potential patterns (themes) and all relevant coded data extracts were coded within the identified themes and sub-themes. This phase was led by DA and completed with AF and RR.
(4) Reviewing themes—themes were reviewed by DA and AF, and broader code groups were created for each theme and entered into Microsoft Excel, version 16.0. Any disagreements about the codes used were discussed among all four investigators (DA, AF, RM, and RR).
(5) Defining and naming themes—DA and AF re-coded the themes and sub-themes, then extracted and detected the story that each theme told and considered whether it fit into the broader context of our data. Each sub-theme was given a final name.
(6) Producing the report—the final themes were analyzed and synthesized into results that were presented in a final report, reviewed by RR and RM.

We followed Tracy's (31) accepted criteria for qualitative best practices, which we have used previously. Transparency was maintained throughout the process of sorting, choosing, and organizing data. The rigor of data analysis was achieved through the development of a rational framework to transform and organize raw data into the research report. Two investigators (DA and AF) analyzed the data and shared it with the rest of the research team to ensure triangulation. Finally, the information was continuously shared with team members during the analysis, with their input based on their various types of professional expertise, strengthening the credibility of the analysis.

## Results

### Observations

We used data from 2,184 surgical cases. Most were general surgeries (37.5%), and most lasted for 1–2 h (53.3%). At the three surgical phases observed, three physicians (SD 0.9–1.02) and two nurses (SD 0.52–0.58) were present (Table 1).

**Table 1:** Characteristics of surgeries observed.

**Characteristic**		**Observations, number, and percentage of total surgeries (*N* = 2,184)**
Surgical specialty	General surgery	820 (37.5%)
	Orthopedics	431 (19.7%)
	Gynecology	239 (10.9%)
	Otolaryngology	216 (9.9%)
	Urology	177 (8.1%)
	Plastic surgery	89 (4.1%)
	Vascular surgery	58 (2.7%)
	Cardiology	55 (2.5%)
	Ophthalmology	51 (2.3%)
	Neurosurgery	39 (1.8%)
Duration of surgery^*^	>1 h	361 (16.5%)
	1–2 h	1,164 (53.3%)
	2–3 h	196 (9%)
	3–4 h	360 (16.5%)
	>4 h	103 (4.7%)
Number of physicians present at the surgical phase (mean ± SD)	Time out	3.28 ± 0.97
	First surgical count	3.02 ± 1.02
	Second surgical count	3.18 ± 0.90
Number of nurses present at the surgical phase (mean ± SD)	Time out	2.30 ± 0.57
	First surgical count	2.29 ± 0.58
	Second surgical count	2.22 ± 0.52

^*^Duration of surgery is represented in categories of hours, 1 min differentiates between categories.

SD, standard deviation.

### Preoperative and intraoperative teamwork

The effects of the preoperative variables on intraoperative teamwork, based on the results of the multivariate binary logistic regression model, are shown in Table 2. The variables tested (amount of preoperative teamwork in the sign-in and time-out phases and the effect of staff presence and turnover on teamwork) predicted a lack of teamwork [$\chi_{\left(6\right)}^{2}$ = 408.110, *p* < 0.0001, Nagelkerke's *r*^2^ = 0.236]. When testing for multicollinearity, none of the independent predictors' variance inflation factor (VIF) exceeded 1.25, supporting the absence of collinearity. There were no significant differences in relation to hospital location (*p* > 0.05) or size (*p* > 0.05).

**Table 2:** Results of the binary logistic regression predicting a lack of teamwork throughout a surgery.

**Variable**	**Odds ratio**	**95% CI for odds ratio**		***p*-Value**
		**Lower**	**Upper**	
Lack of teamwork at preoperative sign-in	1.972	1.741	2.233	<0.001
Lack of teamwork at preoperative time-out	2.142	1.879	2.441	<0.001
Mean number of physicians participating in the surgery	0.830	0.726	0.950	0.007
Mean number of nurses participating in the surgery	0.798	0.642	0.992	0.042
SD of the number of physicians participating in the surgery (turnover)	1.258	1.001	1.580	0.049
SD of the number of nurses participating in the surgery (turnover)	1.227	0.985	1.528	0.068

SD, standard deviation.

Regarding preoperative variables, the effect of each incidence of not performing a sign-in almost doubled the chances of a lack of teamwork when the second surgical count was performed during surgery [odds ratio = 1.972, *p* < 0.001, 95% confidence interval (CI) 1.741, 2.233]. A similar effect was found for not performing the preoperative time-out (odds ratio = 2.142, *p* < 0.001, 95% CI 1.879, 2.441).

The variable of consistent staff presence in the OR revealed a “protective” effect of a minimum mean absolute number of staff and a “harmful” effect of staff turnover during the surgery. Each increase in the number of physicians or nurses decreased the chance of a lack of teamwork by 21 and 24%, respectively (*p* < 0.05). However, each increase in the turnover of physicians reduced the chance of teamwork by 73%. A similar but non-significant trend was seen with the turnover of nurses (*p* = 0.068). There was no significant difference in the results in relation to a hospital's size or location.

### Semi-structured interviews

We interviewed 25 clinicians and risk managers who held administrative roles (Table 3). Most were female with more than 30 years of experience. The interviewees were not observed during the quantitative observations of safety standards.

**Table 3:** Characteristics of interviewees.

**Characteristic**		**Respondents, *N* (%) (*N* = 25)**
Sex	Male	10 (40%)
	Female	15 (60%)
Profession	Anesthesiologist	6 (24%)
	Surgeon	3 (12%)
	Nurse	9 (36%)
	Risk manager (physicians and nurses)	7 (28%)
Experience in profession, years	1–9	0 (0%)
	10–19	5 (20%)
	20–29	7 (28%)
	30–39	10 (40%)
	>40	3 (12%)
Experience in current position, years	0–4	9 (36%)
	5–9	9 (36%)
	10–14	2 (8%)
	15–19	1 (4%)
	20–25	4 (16%)

We identified four main themes regarding the relationship between teamwork and patient safety and staff psychological safety: (1) perception of individual role vs. collaborative team role; (2) team leadership; (3) team characteristics (designated team and team communication); and (4) recommendations to improve teamwork. These themes are expanded upon below.

#### Individual vs. collaborative role

Most physicians and nurses viewed patient safety as their individual responsibility and not that of the team. Most nurses with more than 10 years of experience perceived themselves to be the safety supervisor during a surgery. Their comments included: “We are in charge of implementing the standards in the OR. We supervise how they are performed.” and “Nurses have a huge responsibility. They stop dangerous work processes before harming the patient.”

A surgeon, however, thought that nurses' supervisory role negatively affected their relationship with a surgeon and thus affected the safety and success of a surgery: “Nurses are not nurses anymore. They are a control system that controls and criticizes physicians. They check us all the time. Instead of focusing on their nursing role, they sit and write what the physicians are doing instead of helping them.”

Anesthesiologists' opinions differed. Most viewed themselves as individual safety supervisors: “This is the essence of our role. To assess and evaluate the work environment all the time and make sure everything is working properly.” “Often, I inform the surgeon about relevant background diseases that his patient has. I don't think this is my role, but I see myself as a gatekeeper.” Only a few considered their role to be collaborative: “The safety standards define specific roles for each clinician, but also define our role as a team.”

#### Team leadership

Most interviewees suggested that surgeons should function as team leaders, thereby directing the safety of the surgery. An anesthesiologist stated that “If the surgeons understand that they are in charge of all aspects of the surgery, it will improve safety.” The nurses agreed and added that one meaning of leadership is taking responsibility. “Surgeons don't understand their responsibility. They are supposed to call for a time-out process, but they do not, so the nurses take charge and do it instead.” “When we (nurses) do the surgical count, we know the surgeon needs to be involved and it seems like we bother him.” On the other hand, an anesthesiologist did not think they should be as involved as the nurses: “It is the surgeon's business if he skips the standards and takes shortcuts, I don't deal with it.”

Only a few surgeons, from small rural hospitals, viewed their role to be that of a leader in prioritizing safety standards. “We are performing the surgery and we know what is important and how to prevent errors. Nurses are stricter in following the standards and rules.” “Most of the standards do not focus on risk reduction and can lead to more errors; we know what to focus on.” A risk manager explained that this attitude among surgeons arises from their training: “Surgeons trust shortcuts because they learned in medical school to diagnose the quickest way and then to provide solutions to errors without basing them on standards and checklists.”

A few risk managers explained that surgeons lead a surgery in clinical terms, but not as team leaders. “Their weak point is their hubris. They don't think they should review what others (nurses and anesthesiologists) did. It is like wearing a seat belt when you drive, wearing eyeglasses when you are nearsighted.” For example, “when there is a discrepancy in the count, the surgeon prefers to finish the surgery without waiting for the nurses to recount.”

#### Team characteristics

Two main team characteristics related to safe teamwork were described: working in a fixed, designated team for a specific type of surgery and interprofessional communication.

A designated team was perceived as increasing the team's commitment to the safety of a surgery. A few surgeons thought that this type of team would increase nurses' commitment. “We never leave the surgery in the middle, but stay beyond our shift because this is the right thing to do for the safety of the surgery and the patient. Nurses, however, leave for their lunch break or go home. We have a substitute nurse, but she comes in the middle and does not know what happened before. If the nurses were committed like us and stayed from the beginning to the end, the teamwork would be better and there would be fewer errors.” On the other hand, a nurse described the turnover of surgeons as a factor affecting patient safety. “The surgeon says the surgery is urgent, but leaves for his private clinic in the middle and gets replaced, or he tells me: if you don't prepare the patient to start the surgery before 3 p.m. we will not operate.”

Most anesthesiologists agreed that working in a designated team would benefit the quality and safety of a surgery. “Working in the same team all the time, without turnover, will promote the safety and success of the surgery. When you work with the same people, you know what they think and how they operate.” “If we all work together on the same mission from the beginning of the surgery until the end, we will be able to provide quick responses to urgent issues and consult with each other.”

Communication was mentioned as an essential aspect of teamwork and safe surgery. Most anesthesiologists and nurses emphasized the importance of communication: “The physician and the nurse should communicate well and be involved in each other's work because they work together on a big mission.” “During the sign-in and the time-out, the communication between all staff involved is much better than expected and prevents errors.” “In the OR, we are a multidisciplinary team that works closely together, physically and emotionally, and we have to find a way to interact and communicate effectively.”

An anesthesiologist noted that poor communication between surgeons and anesthesiologists can affect patient safety: “It is very rare that there are errors in machines and equipment; the main errors are related to decision-making and lack of communication between us. For example, something went wrong in the surgery but the surgeon did not think to call the anesthesiologist who was around and could assist.” Interestingly, one surgeon noted that: “There should be communication between the patient, anesthesiologist, and surgeon during the surgery.”

Inappropriate communication can be hurtful and may even deteriorate into bullying that can risk the staff's psychological safety. Some nurses described situations in which they were bullied by physicians: “I tell the surgeon that I am missing a sponge in the count, who screams that I should go to school and learn how to count. So, I insist on stopping the surgery and refuse to give him the stitches to close the fascia…In the X-ray, the sponge was found behind the heart… I feel like I am in a warzone.” “There was a discrepancy in the surgical count, but the surgeon insisted that everything was OK. I stepped in and told him that I am the supervising nurse, and I will call his manager if he does not stop the surgery. He stopped and the sponge was found in the urethra.”

#### Recommendations for improving teamwork

Most physicians and nurses suggested performing simulation training in controlled settings to improve teamwork. A surgeon suggested “a controlled simulation of interdisciplinary teamwork that would include training in leadership and communication skills.” A nurse suggested that the simulation should include “performance of safety standards and communication skills, such as speaking up and conflict management.” A risk manager suggested implementing interdisciplinary root cause analysis after any adverse events. “Performing root cause analysis by the OR staff will enable discussing teamwork issues freely and resolving them without concerns due to the presence of risk management or hospital administrators.” “It will lead to trust among the team members and better solutions that will prevent future errors.”

Surgeons, anesthesiologists, and nurses all thought that technological solutions would facilitate their work processes and promote a better work environment. Some surgeons suggested using a digital time-out adjusted to patients' requirements that would reflect the risks related to the particular patient and surgery. Anesthesiologists recommended computerized systems that would integrate patient data and signal an alert regarding anesthesia risks. Nurses thought that scanners would ease the surgical counting process.

## Discussion

Teamwork is an essential component of risk reduction, patient safety, and staff psychological safety during a surgery and contributes to preventing Never Events. For this study we analyzed interprofessional preoperative teamwork and its effect on intraoperative teamwork; we then identified factors affecting teamwork that are related to patient safety and staff psychological safety.

The results revealed that teamwork in the preoperative setting and consistent staff presence during a surgery, without turnover, were predictors of teamwork during surgery. A few studies have evaluated preoperative teamwork but not in relation to teamwork during surgery or to risks to patient safety, as analyzed here. Myklebust et al. (32) described the preoperative phase as busy, because each clinician must complete preparatory tasks as quickly as possible to prepare the patient, which can be a chaotic process when trying to simultaneously accomplish individual and collaborative tasks. This can lead to conflict and an unpleasant atmosphere, which was also supported in our findings that staff perceived their role as individuals rather than a team and led to challenges in team communication. Although we did not find any studies that directly evaluated the effect of preoperative teamwork on intraoperative teamwork in relation to safety standards, it is likely that preoperative tension might continue during a surgery and inhibit the key determinants of staff psychological safety: speaking up, team collaboration, and experimentation (33).

Another predictor of teamwork during surgery is the number of team members. We found that that additional physicians and nurses increased the degree of teamwork. We did not find any studies that had defined an adequate number of staff members needed on a surgical team or their composition per specific surgery. However, some studies did find that adequate surgical team size had a positive effect on teamwork, possibly because there are more people available to help complete tasks and share the total cognitive load (34, 35). Adequate staffing can compensate for unexpected emergencies or prolonged surgical cases (36). Inadequate staffing has been identified as a barrier to teamwork, mostly by nurses and surgeons and to a lesser extent by anesthesiologists (14). In contrast, however, a few studies have found that larger teams might create barriers to optimal performance because of the greater communication demands and role ambiguity (12), which may prolong operative time (37).

Regardless of the required team size, the staff we interviewed highlighted the importance of a permanent, designated team, which reinforced our findings regarding turnover. Staff turnover during a surgery was considered to have a negative effect on teamwork, was perceived to show a lack of commitment, and caused the risk of a breakdown in communication due to the lack of familiarity among team members and with a patient's condition. Nursing turnover during a surgery was found to increase opportunities for breakdowns in communication during handover (38), as it interrupts the flow of surgery (10) and may prolong it (39). A review found that anesthesiologists usually take breaks as part of their work culture, but they are aware of the importance of handoffs in relation to patient safety. However, surgeons rarely take breaks, as they feel that leaving a surgery would affect its success (40).

One suggestion for improving teamwork arising from our study included working in a fixed, designated team that is led by the surgeon. Surgical teams are often constructed on an *ad hoc* basis and thus fluctuate, which can lead to a lack of familiarity (13). Familiarity enables a shared definition of teamwork and professional roles that can increase positive surgeon–anesthesiologist relationships (14, 34). Doll et al. (41) found that a managerial decision to assign a particular anesthetist to a surgeon and to use a predefined surgical list resulted in decreased operative times. This may be because a team in which each clinician has confidence in her or his colleagues and works on the basis of common principles and values can work more quickly while still avoiding risks to patient safety (11). Such confidence among team members can be achieved through teamwork training in soft skills, such as communication, which was found to be the primary means to increase coordination among healthcare team members (42). Evidence in the literature regarding who should lead a surgical team is sparse. Some have assumed that the surgeon is the leader (36), but others have assumed that it could be anesthesiologists due to their perioperative role in standardizing patient care and leadership (13).

Our interviewees described communication as an essential component of teamwork. In general, effective team communication improves patient outcomes and prevents errors (43). Safety risks can be identified and responded to by conducting a daily huddle aimed at preventing specific safety risks due to surgical errors (44).

Our findings revealed the existence of ineffective communication between surgeons and anesthesiologists, which may affect clinical decision-making and patient safety. Possible explanations for this ineffective communication include ineffective interprofessional communication (45) and differing mental models and role perceptions (16).

According to our findings, there was some disrespectful communication between surgeons and nurses. In an earlier survey of 7,465 clinicians, 70.1% had experienced incivility and 36.9% had been bullied (46), which may inhibit individuals from speaking up and prevent the maintenance of a psychologically safe team (22). The reasons suggested included intrapersonal (personality traits, psychological conditions, transient psychological states), organizational (production pressures, mismanagement, administrative inefficiency, working conditions), and interpersonal (perception of status, hierarchy, situational triggers) (46, 47).

### Strengths and limitations

The strengths of this study include that it revealed new insights into teamwork in the OR, specifically in relation to safety. The mixed methods design allowed us to obtain a comprehensive picture of the effect on teamwork of performing safety standards such as a surgical safety checklist and surgical counts. We also explored factors contributing to or preventing teamwork during surgery that could risk patient safety and a team's psychological safety.

The main limitation of this study was the inability to control the methodology used to collect the observational data received from the MOH, which limited our ability to analyze personal and environmental factors that may affect teamwork. Factors such as gender, areas of expertise, and the length of experience of clinicians observed were lacking, so as the workload at the OR at the time of observation. The data also lacked information about other team members who may affect teamwork, such as technicians. The data represent observations performed over 4 years, which may affect the generalizability of the findings.

## Conclusion

This study revealed that the level of preoperative teamwork can predict the level of intraoperative teamwork, specifically with regard to patient safety. We also found that the number of clinicians participating in a surgery and their level of turnover affects teamwork. Factors that would support effective teamwork are designated teams with defined roles and having leaders who promote teamwork and effective communication. An effective team is key to boosting the teamwork and team engagement that is associated with improved team performance in relation to staff psychological safety and patient safety.

We recommend promoting the psychological safety of medical staff by mediating between the requirements of individual professional roles and the expected collaborative team roles and the work environment in the OR. This could be accomplished by creating fixed, designated surgical teams with a defined leader who manages all aspects of a surgery and its teamwork. This will promote patient safety and staff psychological safety. The team members should have sufficient familiarity with each other to solve problems, engage in mutual learning from errors, and improve safety. These teams would benefit from soft-skills training that can increase their coordination and engagement through routine team huddles. An advanced technological environment that facilitates work processes for the team could also benefit their performance. Further study is needed to define the appropriate size and composition of a surgical team needed to ensure patient safety in every surgical procedure through ensuring effective teamwork that promotes staff psychological safety.

## Data availability statement

The original contributions presented in the study are included in the article/Supplementary material, further inquiries can be directed to the corresponding author.

## Ethics statement

Ethical approval for the study was obtained from the Medical Research and Ethical Committee of the Israeli Ministry of Health (MOH 032-2019), on 27 December 2019. The need for informed consent was waived because only deidentified data were used. The individuals interviewed provided verbal consent to participate and received no compensation.

## Author contributions

All authors participated in developing the initial idea and design of the study, data collection, analysis and interpretation, writing the initial draft and critically revising the manuscript, and take responsibility for the accuracy of the material contained in the study. All authors contributed to the article and approved the submitted version.
