# Supplementary material for: Patient safety and staff psychological safety: A mixed methods study on aspects of teamwork in the operating room
Source: Front Public Health. 2022 Dec 23;10:1060473. doi: 10.3389/fpubh.2022.1060473 (PMC9816421; doi:10.3389/fpubh.2022.1060473)
Supplement: Supplementary file 1 [file Table_1.docx]

**Appendix 1. Structured observation of items representing teamwork throughout a surgery**

**Preoperative: Surgical Safety Checklist**

***Sign-in phase***

| **N/A** | **No** | **Yes** | **Statement** |
| --- | --- | --- | --- |
|  |  |  | Sign-in performed by surgeon, anesthesiologist and nurse |
|  |  |  | Signature (surgeon, anesthesiologist, nurse) |

***Time-out phase***

| **N/A** | **No** | **Yes** | **Statement** |
| --- | --- | --- | --- |
|  |  |  | Sign-in performed by all staff members present in the operating room |
|  |  |  | Time-out is performed by staff members before surgical cut |
|  |  |  | All staff members stop their activity and listen to the time-out |
|  |  |  | Verbal agreement of all staff members to details of the time-out |
|  |  |  | Signature of all staff members |

**Intraoperative: Surgical count**

***Second surgical count - closure of fascia/cavity is initiated***

| **N/A** | **No** | **Yes** | **Statement** |
| --- | --- | --- | --- |
|  |  |  | Surgical Count is performed by scrub nurse and circulating nurse |
|  |  |  | Surgeon announces to nurses his intention to close the fascia/cavity before its actual closure in order for the nurses to start counting |
|  |  |  | Surgical Count is performed by two nurses when surgeon announces intention to close the fascia/cavity |
|  |  |  | Surgical count is made out loud by two nurses with the participation of all other staff members (surgeon and anesthesiologist) |
