# Supplementary material for: Patient safety and staff psychological safety: A mixed methods study on aspects of teamwork in the operating room
Source: Front Public Health. 2022 Dec 23;10:1060473. doi: 10.3389/fpubh.2022.1060473 (PMC9816421; doi:10.3389/fpubh.2022.1060473)
Supplement: Supplementary file 2 [file Table_2.docx]

**Appendix 2. Semi-structured interview**

**Key informant interview guide**

**INTERVIEW LOGISTICS**

| Interview Date (month/day/year) |  |
| --- | --- |
| Interviewer |  |
| Duration of Interview (minutes) |  |
| Additional Notes |  |

**INTERVIEW QUESTIONS**

| Part I: General information about your work position 1. How would you describe your main role at Ministry of Health (MOH)/hospital?  2. Type of clinician: Physician Nurse Other  3. Administrative status: Do you have an administrative role in the MOH/hospital?  Yes  No  <1 1-4 5-7 8-10 11-15 16-20 >21  4. Years of MOH/hospital experience: |
| --- |
| **Part II: Attitude towards “Never Events” in operating rooms in Israel** |
| Now I would like to focus on your attitude towards Never Events in the operating rooms in Israel  5. How would you define Never Events in the operating rooms?  PROBE: Are there different types of Never Events in the operating rooms?  PROBE: Preventable vs. Not Preventable  6. In general, to what extent do you feel that Never Events are a real safety issue in the operating rooms?  7. Based on your experience, how frequent are Never Events in the operating rooms?    8. Based on your experience, what are the main causes of Never Events in the operating  rooms?  PROBES: On different levels; system-level factors, individual factors  9. Does MOH/your hospital (i.e., operating department) utilize a structured interventional program to eliminate Never Events in the operating rooms? If yes, please elaborate  10. Do you personally remember any targeted actions that were conducted in MOH/your hospital (i.e., operating department) to eliminate Never Events in the operating Rooms? If yes, please elaborate?  11. How comfortable are you reporting issues related to Never Events in the operating room to your manager/administration?  PROBES: How comfortable are your colleagues? |
| **Part III: Personal experience with “Never Events” in the operating room** |
| 12. Were you exposed to a Never Event in the operating room? If yes, can you please tell me what happened?  PROBES: In your opinion, what were the main causes of the Never Event in this case?  PROBES: Do you think the Never Event in this case was preventable?  PROBES: Do you have any suggestions for how to avoid a case like that in the future?  13. Any other comments you have about this case? |
| **Part IV: Suggestions for innovative tools or processes to reduce “Never Events”** |
| 14. To what extent do you think that innovative solutions can help to eliminate Never Events in the operating room?  PROBES: Care processes, educational sessions, IT solutions  15. Any suggestions for innovative tools or processes to eliminate Never Events in the operating room?  16. Do you think that predictive analytics solutions could predict potential Never Events in the operating room?  17. In general, what other suggestions or comments might you have for us? |

Thank you for participating. Your opinion and input are very appreciated.

THANK YOU VERY MUCH.
